# Suture type used for perineal injury repair and sexual function: a randomised controlled trial

**DOI:** 10.1038/s41598-020-67659-2

**Published:** 2020-06-29

**Authors:** Juan Miguel Martínez-Galiano, Beatriz Arredondo-López, Manuel Hidalgo-Ruiz, Alicia Narvaez-Traverso, Inmaculada Lopez-Morón, Miguel Delgado-Rodriguez

**Affiliations:** 1 Department of Nursing, University of Jaen, Jaén, Spain; 2 CIBER Epidemiology and Public Health (CIBERESP), Madrid, Spain; 3 Andalusian Health Service, Andalucia, Spain; 4 Department of Public Health, University of Jaen, Jaén, Spain

**Keywords:** Health care, Signs and symptoms

## Abstract

The type of suture used to repair perineal injury may be associated with this healing process and subsequent sexual function. This study aims to assess whether the suture technique used (continuous or interrupted) has an impact on a woman’s sexual function following childbirth. A single-blind randomised clinical trial was conducted with primiparous women who had experienced a perineal injury during childbirth. A computer-generated random number table was applied to allocate women to each group. Data were collected on sociodemographic variables, variables associated with childbirth, and outcomes during the 3 months after childbirth. Mean difference was used to assess the influence of the suture type on outcomes. Multivariate analyses were carried out to adjust for unbalanced variables after randomisation. Seventy women participated in the intervention group (continuous suture) and 64 women in the control group (interrupted suture). The women in the intervention group scored high for sexual desire, adjusted mean difference (aMD) = 1.8, 95% CI = 1.1–2.6 (p < 0.001); the same happened with arousal (aMD = 1.7, 95% CI = 0.8–2.5, p < 0.001). In the intervention group, orgasm was more easily reached, aMD = 0.8, 95% CI = 0.4–1.1 (p < 0.001). Women who received a continuous suture indicated they felt less discomfort (p < 0.001). Women who had a continuous suture reported better postpartum sexual function.

**Trial registration:** ClinicalTrials.gov NCT03825211 posted 31/01/ 2019.

## Introduction

An injury to the perineum may occur during childbirth. This injury may be produced spontaneously on expulsion of the foetus—tear—or be made by the healthcare professional attending the birth—episiotomy^1,2^. The majority of women experience some short-term discomfort or pain following repair of the perineal injury, and up to 20% continue having problems in the long-term, for example, slight dyspareunia^3^.

The World Health Organisation considers sexuality a central aspect of the quality of life of a woman, and it reflects her physical, psychological, and social well-being^4^. Questionnaires are available, such as that of Sánchez et al.^5^, to evaluate the sexual function of women. The instrument by Sánchez et al. evaluates the phases of sexual response, initiative, and the degree of sexual communication. It also collects descriptive data on sexual performance and is useful in the exploration and diagnosis of sexual dysfunctions. Another questionnaire used for this purpose is the “Female Sexual Function Index” developed by Rosen et al.^4^. This is an instrument that meets the classification of the International Consensus Development Conference on Female Sexual Dysfunctions. It is self-administered, reliable, and straightforward to evaluate female sexual function in a wide age range, and has demonstrated its reliability in the evaluation of female sexual function^4^. A healthy sexual life has numerous benefits^5^.

Among the most common sexual dysfunctions that women may suffer from are the lack of libido, the inability to reach orgasm, sexual pleasure, and pain during sexual relations^4,6–8^. The puerperium is a critical phase for the onset and increase of these sexual dysfunctions^9–12^, and the deterioration of sexual function is one of the potential long-term sequelae following childbirth associated with a perineal injury^9^. The repair of this injury may also influence the incidence of sexual problems, although results to date have been inconsistent^3,13–17^. Two randomised clinical trials have been conducted in Denmark comparing several suture procedures^15,16^: lower rates of dyspareunia were reported in the experimental groups with continuous sutures, but these were only significant in the Detlefsen et al. trial^15^. In 2008, a randomised controlled trial was conducted in Spain, with 223 women receiving a continuous suture and 222 women having interrupted sutures, without any significant differences found between the two techniques in terms of dyspareunia^13^. In Turkey, in 2009, a study was conducted with 160 women, which concluded that there were no statistically significant differences between the groups in the proportion of women that recommenced sexual relations early^14^. In 2009, a study was conducted in Pakistan in which 200 patients were included who had either an episiotomy or second-degree perineal tear, using both suture techniques and different suture materials, finding the same results in terms of dyspareunia in the groups investigated^17^. In a Cochrane revision of 16 studies conducted in 2012 by Kettle et al.^3^ the final recommendation was to conduct further studies on the suture used in the repair of the perineum considering those results most important to women, including postpartum sexual problems^3^.

The technique used in the repair of the perineal lesion may avoid alterations in female sexual function following childbirth. Existing studies show conflicting results and encourage more research^3,13–17^. For these reasons, we proposed the research objective to evaluate whether the type of suture (continuous or interrupted) has repercussions on the sexual function of a woman following childbirth.

## Results

Seventy women were included in the group in which the women received continuous suture (intervention group), and 64 in the group where women interrupted sutures was used (control group). Of all variables in Table 1, significant differences were found for only two variables (civil status and attendance at a health education programme during pregnancy). Women who received a continuous suture had a higher percentage of married women than in the group of women who had an interrupted suture, 67.2% vs. 59.4%, respectively (p = 0.047). 54.3% (n = 38) of the continuous suture group attended an education program during pregnancy, and the percentage was 73.4% (n = 47) in the interrupted suture group (p = 0.031).

**Table 1:** Description of the study population.

Variables	Continuous suture *n* (70)	Interrupted suture *n* (64)	*p* value
**Age** (years), m (SEM)^a^	30.98 (0.63)	30.76 (0.68)	0.813
**Civil status**			0.047
Single, *n* (%)	7 (10.00)	17 (26.56)	
Married, *n* (%)	47 (67.14)	38 (59.38)	
Stable relationship, *n* (%)	15 (21.43)	9 (14.06)	
Divorced, *n* (%)	1 (1.43)	0 (0.00)	
**Education level**			0.797
Incomplete primary, *n* (%)	2 (2.86)	1 (1.56)	
Primary, *n* (%)	3 (4.29)	3 (4.69)	
Incomplete secondary, *n* (%)	2 (2.86)	4 (6.25)	
Secondary, *n* (%)	17 (24.29)	18 (28.13)	
Higher secondary education, *n* (%)	19 (27.14)	12 (18.75)	
University, *n* (%)	26 (38.57)	27 (40.63)	
**BMI**^b^, m (SEM)	28.19 (0.51)	27.27 ( 0.46)	0.185
**Previous illness, yes,** *n* (%)	6 (8.57)	8 (12.50)	0.575
**Exercise during pregnancy, yes,** *n *(%)	39 (55.71)	34 (53.13)	0.862
**Perineal massage pre-labour, yes,** *n* (%)	15 (21.43)	12 (18.75)	0.830
**Health education during pregnancy, yes,** *n* (%)	38 (54.29)	47 (73.44)	0.031
**Length of gestation (weeks),** m (SEM)	39.57 (0.13)	39.78 (0.14)	0.275
**Start of labour**			0.473
Spontaneous, *n* (%)	48 (68.57)	40 (62.50)	
Induced, *n* (%)	22 (31.43)	24 (37.50)	
**Epidural analgesia, yes,** *n* (%)	56 (80.00)	53 (82.81)	0.825
**Duration of dilation** (min), m (SEM)	314.80 (22.87)	342.72 (21.10)	0.373
**Duration of pushing** (min), m (SEM)	85.61 (6.29)	96.64 (8.23)	0.284
**Duration of delivery** (min), m (SEM)	9.71 (1.06)	9.70 (1.02)	0.994
**Type of perineal lesion**			
Episiotomy, *n* (%)	32 (45.71)	35 (54.69)	0.387
2nd degree tear, *n* (%)	38 (54.29)	29 (45.31)	
**Weight of newborn** (g), m (SEM)	3,252.6 (41.8)	3,185.1 (50.5)	0.301

^a^m (SEM): mean (standard error of the mean).

^b^BMI (body mass index) = kg/m^2^.

The number of women who resumed sexual intercourse by 15 days was small; 4.29% (3) in the continuous suture group versus 1.56% (1) of the interrupted, however, the difference was not significant (aOR = 4.14, 95% = 0.36–47.91; p = 0.256). At 3 months, the number of women who normalised their sexual relations was higher in the intervention group, 71.01% (49) versus 37.10% (23) from the interrupted suture group (aOR = 4.78, 95% CI = 2.14–10.64; p < 0.001). There were no significant differences regarding the number of days after childbirth to restart sexual intercourse with coitus, although it was higher in the control group: an average of 50.8 ± 2.3 days in the interrupted suture group, compared to 46.2 ± 2.2 days intervention group (p = 0.168).

The differences in the mean score of several sexual functions are displayed in Table 2. Women in the intervention group had higher sexual desire scores in the control group, with an adjusted mean difference (aMD) = 1.8, 95% CI = 1.0–2.6, p < 0.001. With regards to sexual excitation, having a continuous suture was also a protective aspect compared to the interrupted suture group (aMD = 1.7, 95% CI = 0.8–2.5, p < 0.001). Women in the continuous suture group lubricated better than those in the interrupted suture group (aMD = 0.7, 95% CI = 0.3–1.1, p < 0.001). It can also be appreciated how achieving orgasm more easily was another of the aspects which scored higher in the intervention group in comparison with the interrupted suture group (aMD = 0.08, 95% CI = 0.4–1.1, p < 0.001). Regarding pain experienced in sexual intercourse with penetration, women who received the continuous suture scored higher figures than those who had interrupted sutures (aMD = 1.3, 95% CI = 1.1–1.5, p =  < 0.001); indicating that those in the intervention group felt less discomfort. In terms of anticipatory anxiety for sexual intercourse, those in the continuous suture group achieved higher values than those in the interrupted suture group, with an aMD = 0.3, 95% CI = 0.1–0.6, p =  < 0.001. The women who had a continuous suture had a higher score for the item of communication than those with interrupted sutures, (aMD = 0.4, 95% CI = 0.1–0.7, p =  < 0.001). Likewise, satisfaction was higher in the intervention group (aMD = 1.2, 95% CI = 0.7–1.7, p < 0.001).

**Table 2:** Evaluation of female sexual function at 3 months postpartum.

Variables	Univariate analysis		Multivariate analysis^a^	
	Mean difference (95% CI)	*p* value	Mean difference (95% CI)	*p* value
Desire or sexual excitation	1.9 (1.1–2.6)	0.001	1.8 (1.0–2.6)	< 0.001
Sexual excitation	1.6 (0.8–2.5 )	0.001	1.7 (0.8–2.5)	< 0.001
Lubrication or vaginal wetness	0.7 (0.3–1.1)	0.001	0.7 (0.3–1.1)	< 0.001
Orgasm or climax	0.7 (0.4–1.1)	< 0.001	0.8 (0.4–1.1)	< 0.001
Dyspareunia	2.2 (1.4–3.0)	< 0.001	2.2 (1.3–3.1)	< 0.001
Anxiety	0.2 (0.1–0.6)	0.162	0.3 (0.1–0.6)	< 0.001
Pain	1.2 (1.1–1.5)	< 0.001	1.3 (1.1–1.5)	< 0.001
Communication	0.4 (0.1–0.6)	0.008	0.4 (0.1–0.7)	< 0.001
Satisfaction with sex life in general	1.1 (0.6–1.6)	< 0.001	1.2 (0.7–1.7)	< 0.001

^a^Variables adjusted in the multivariate analysis: civil status and attendance to a health education programme during pregnancy.

## Discussion

The women who had a continuous suture showed a clear improvement in sexual intercourse. Not only did women with continuous suture have a higher frequency of normalisation of their sexual relationships by 3 months after childbirth, but most of the aspects of sexual relationships (lubrication, climax, desire, dyspareunia, etc.) also improved clearly in this group.

A strength of this study is that the woman did not know what type of suture had been used (simple blind); hence their responses could not be influenced by knowing their intervention. In the exclusion criteria, parity was considered as it influences the discomfort and problems in the postpartum period^18^. The exclusion of women who had not had an eutocic delivery was to avoid the influence of the type of delivery on the results. The type of delivery has been associated with the presence of sexual problems in the puerperium^19^. Among the limitations of this study is the sample size, which prevented the random allocation functioning completely, and some variables were not correctly balanced between the two groups. This was the case for civil status and attendance at a health education programme during pregnancy. This imbalance was corrected for in multivariate analyses, which did not modify the results of the crude analysis. Another limitation that may exist is that the midwives who collected the data knew the study group to which the women belonged, which could affect the collection of data on sexuality. The suturing personnel should fulfil the following four criteria: (1) have completed a training course on the continuous suturing technique, (2) show at least 1 year of experience in performing this technique, (3) have a minimum experience of 5 years in assisting deliveries, and therefore in suturing perineal injuries, and (4) accept participation in the trial. With these criteria only 5% (1) of the midwifery staff of the Torrecárdenas hospital participated, 10% (2) in the Jaén hospital complex, 12% (3) in Granada, and 20% (2) in both the Úbeda and Linares hospitals (a total of 10 trained midwives). Therefore, it was necessary to include several centres. In addition, its multi-centre character showed an advantage: its increased external validity after assessing that the results were similar in the different hospitals.

Gómez Cantarino and Moreno Preciado^20^ concluded that pregnancy and childbirth is experienced differently for each woman, which could affect her life and sexuality in diverse ways. The ascertainment of 'experience' required a qualitative approach and was not used in our study.

Several published studies relate perineal injury with maternal morbidity in the postpartum period, but they do not address sexual dysfunction with the type of suture used to repair the perineal lesion^3,21–27^. In the United States of America, Pauls et al.^28^ observed that female sexual dysfunction is a common health problem that affects approximately 43% of women. This may be affected in one way or another depending on the type of perineal injury caused by childbirth and the technique used to repair it. In general, in our study, the women in the continuous suture group had higher scores for desire, arousal, better vaginal lubrication or wetness, and achieved orgasm more easily, than the women in the interrupted suture group. Different studies have analysed sexual function, such as Barrett et al.^29^ in the United Kingdom, Morano et al.^30^ in Italy, Almeida et al.^31^ in Brazil, Perveen et al.^17^ in Pakistan, Valenzuela et al.^13^ in Spain, and a meta-analysis by Kettle et al.^3^. The results of all these studies agree: There were no differences between the incidence of dyspareunia and the type of suture used in the repair of the perineum.

Kokanali et al.^14^ observed that there were no significant differences between the type of suture used and the recommencement of sexual intercourse. These results contrast with those found in our study, in which it can be seen how the normalisation of sexual relations at 3 months after birth was more frequent in the group with a continuous suture than in the group of women who had an interrupted suture.

Although sexual intercourse was restarted 5 days a week earlier in the group of women with a continuous suture, the difference between both groups was not statistically significant, which is in line with the study by Kokanali et al.^14^. Valenzuela et al.^13^ and Shrivastava et al.^32^ found opposite results, with a later restart of sexual intercourse in the continuous suture group. Our results show that women with interrupted sutures are at higher risk of not having normalised their sexual relations by 3 months after birth, consistent with the findings of Detlefsen et al.^15^ and Isager Sally et al.^16^.

Amiri et al.^6^ and Barrett et al.^29^ suggested that sexual function in women during the 3–6 months postpartum was significantly lower than in the period prior to pregnancy. Similarly, González Labrador and Miyar Pieiga^33^ said that resuming sexual activity in the puerperium is an important issue in the life of women. Signorello et al.^22^ also noted that the quality of a couple’s sexual life after childbirth is clearly affected. A proven reality is that during the puerperal period, frequency and sexual enjoyment decrease dramatically, and this dysfunction is greater in those puerperal women who have suffered perineal trauma. According to González Labrador and Miyar Pieiga^33^, sexual intercourse can be affected by the healing of the episiotomy or tear. Likely the repair of this type of injury influences its recovery; therefore, a continuous suture technique, which uses less material and has fewer knots, favours wound healing and faster healing, which at the same time may influence the improvement of the sexual function of women after childbirth. This may explain why the women in the continuous suture group of our study normalised their sexual relations earlier, and therefore their sexual function was better than in the control group.

## Conclusion

In conclusion, the findings of our research showed that women undergoing a continuous suture normalised sexual relations earlier, achieved orgasm more easily, and obtained higher scores in sexual communication and satisfaction of sexual life than those in the group with interrupted sutures.

## Methods

A controlled multicentre clinical trial was conducted, with random allocation of two treatment groups, between November 2016 and May 2018.

### Population selection

The reference population was women who gave birth in the following hospitals placed in southeast Spain: San Juan de la Cruz in Ubeda (Jaen), San Agustin in Linares (Jaen), Jaen University Hospital Complex, University Hospital Virgen de las Nieves (Granada) and Hospital Torrecárdenas (Almeria).

The inclusion criteria were as follows: age > 18 years, primiparous, singleton, eutocic delivery, second-degree perineal tear o an episiotomy as part of labour, and a newborn weight between 2500 and 4000 g. The exclusion criteria were: language barrier, problems related to the pelvic floor before labour (prolapse, incontinence, vulva varices, etc.), dyspareunia or sexual dysfunction, or haemorrhoids perceived as uncomfortable or painful.

Ethical approval for this study was obtained from the Ethics Committees of each participating centre in the study: Comité de Ética de la Investigación del Hospital Universitario Virgen de las Nieves de Granada (Committee of Ethics of Investigation of the University Hospital Virgen de las Nieves in Granada), Comité de Ética de la Investigación del Hospital Torrecardenas (the Committee of Ethics of Investigation of the Torrecardenas Hospital in Almeria), Comité de Ética de la Investigación del Complejo Hospitalario Universitario de Jaén; (the Committee of Ethics of Investigation of the University Jaen Hospital) Comité de Ética de la Investigación del Hospital de Úbeda (the Committee of Ethics of Investigation of the Ubeda Hospital) and Comité de Ética de la Investigación del Hospital de Linares (the Committee of Ethics of Investigation of the Linares Hospital). The participating women signed written informed consent, in accordance with the principles of the Declaration of Helsinki (Fortaleza, 2013). Trial registration: ClinicalTrials.gov NCT03825211 posted 31/01/ 2019^27^.

**Figure 1:**
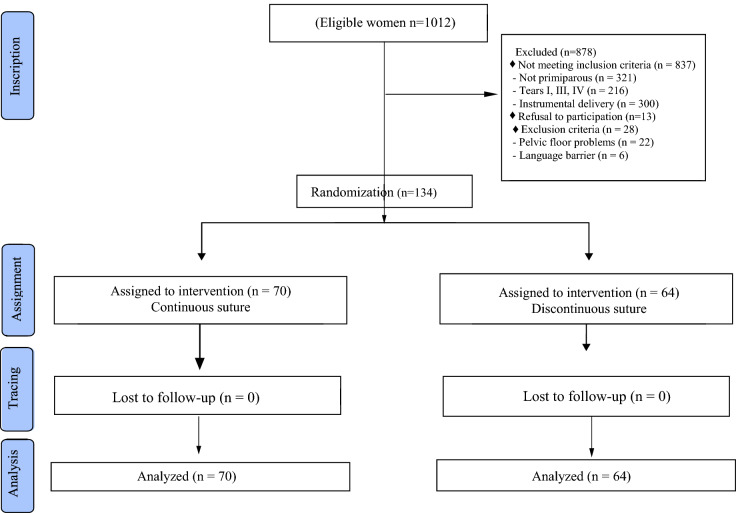
Flow diagram of the participants.

To estimate the sample size, perineal pain in the medium term was taken into account. To detect a difference between 60.4% with pain in the group of women with interrupted sutures and 32.3% in women with a continuous suture^30^ with a statistical power of 80%, an alpha error of 5%, a ratio of 1:1 between the experimental and control groups, 54 women would be needed in each group. Assuming a loss of 20%, the estimated sample size was 67 women in each group (Fig. 1).

### Intervention

Two types of sutures using different techniques were used for perineal injury repairs. Continuous sutures were used in group A and interrupted sutures in group B. The two techniques and sutures are described in more detail in the Supplementary information. A computer-generated random number table was applied to allocate women to each group. The sequence was placed in individual opaque envelopes, which were opened when a woman met the inclusion criteria.

The personnel that performed the sutures had previously taken a training course on continuous sutures and had at least 1 year of experience in these. Additionally, these healthcare professionals had an experience of at least 5 years in attending childbirth and, therefore, in suturing perineal injuries. All sutures were carried out by 10 trained midwives. Only 5% (1) of the midwives working at Torrecárdenas hospital participated, as well as 10% (2) in the Jaen hospital, 12% (3) in Granada, and 20% (2) in Úbeda and Linares hospitals.

### Data collection

Data were collected on sociodemographic information, type of perineal lesion (second-degree tear or episiotomy), type of suture used, initiation of sexual relations. At 3 months postpartum a phone interview was conducted to evaluate sexual function using the validated scale by Sánchez et al.^5^. Women were asked about recovery of sexual relations, their quality level in comparison to before childbirth and pregnancy, and the number of days after postpartum until they had engaged in sexual intercourse with coitus.

A questionnaire was developed to collect the data to be applied by midwives. Midwives interviewed women in the delivery room, informing them of the study objectives, that they will not know the suture technique (blind of participants), and giving an informed consent form. At this point, information was also collected on sociodemographic and obstetric variables. Data were also obtained and verified from the clinical history, the maternal pregnancy record, and the telephone interviews conducted for the follow-up of each case.

### Follow-up

The follow-up of sexuality was conducted in the following manner:

- At 15 days, the women were asked if they had recommenced sexual relations.
- At 3 months, general sexual function was evaluated. In addition, via direct and open questions using the validated scale by Sánchez et al.^5^ women were asked about the normalisation of sexual relations (the same as before childbirth/pregnancy), whether they had engaged in sexual intercourse/coitus, and the number of days postpartum until sexual intercourse.

### Data analysis

The information was introduced in a database created with the programme Epi Info. From there, the data were exported using the programme Stat-Transfer 13 (Circle Systems, Seattle, Washington, USA) to be processed with the programme Stata 15-SE (Stata Corporation, College Station, TX, USA). Data were cleaned via logical cross-field validation to detect impossible values. Statistical analyses were conducted after the elimination of errors.

First, the balance between the groups was assessed for all the variables that could determine a differential response in the woman. Significant differences were found for civil status and for attendance at a health education programme during pregnancy (described in the “Results”). Given that these variables could affect the outcomes, it was decided to conduct analyses adjusting for these two variables. A descriptive analysis of the sample was done using percentages for qualitative variables and the mean (m) ± standard deviation (SD) for continuous variables. The association of the type of suture with each variable was assessed with binary variables (yes/no) using logistic regression, crude, and adjusting for marital status and health education during pregnancy. The relationships between the type of suture and continuous variables were assessed by the crude mean difference (MD) and its 95% confidence interval (CI) of the scores. To adjust for imbalances after random allocation the adjusted MD (aMD) and its 95% CI was calculated by the analysis of covariance.

## Supplementary information

Supplementary file1 (DOC 33 kb)

## Data Availability

The datasets generated during and/or analysed during the current study are available from the corresponding author on reasonable request.
