# Supplementary material for: Suture type used for perineal injury repair and sexual function: a randomised controlled trial
Source: Sci Rep. 2020 Jun 29;10:10553. doi: 10.1038/s41598-020-67659-2 (PMC7324616; doi:10.1038/s41598-020-67659-2)
Supplement: Supplementary file 1 — Supplementary file1 (DOC 33 kb) [file 41598_2020_67659_MOESM1_ESM.doc]

**ANNEX**. Description of the intervention

**Group A. *Continuous suture technique*:**

Suture material: “Safil quick” 2/0, polyglycolic acid, braided, coated, rapid absorption. All parts of the perineal lesion will be sutured with the same suture thread.

* Vaginal mucosa suture: from the apex of the vaginal wound, the first stitch is placed 0.5-10mm above the apex to ensure hemostasis. This suture is secured with a double knot to the right, simple to the left, and finished with a simple to the right. Place a continuous suture from the apex to the hymenal ring. Suture should include the vaginal mucosa and rectovaginal fascia, placed approximately half a centimetre from the edges of the wound. A continuous suture will be placed without crossing the thread.

* Perineal muscle suture: Insert the needle on the left side of the mucosa to introduce the needle deeply in the centre of the perineal muscles. From the centre of the perineal wound, insert the needle and pull through approximately half a centimetre from the edge of the perineal skin on the right side, insert the needle in the left side, and it should emerge deeply in the centre of the perineal wound. Repeat this continuous non-locking suture technique until the perineal muscles are opposing again and the dead space closed.

* Skin suture: the skin should be correctly approximated, but without tension, as a skin suture can increase the incidence of perineal pain in the first postpartum months. Continue from the superficial muscle until the skin is approximated and finish with a subcutaneous/intradermal stitch, burying in the muscle and securing with two knots, one to the right and the other to the left, at a distance of 1-2 cm from the suture.

**Group B. *Interrupted suture technique*:**

* Vaginal mucosa suture: suture material: “Safil quick” 0/0, polyglycol acid, braided, coated, rapid absorption. Identify the wound apex. The anchor point of the suture should be 1cm above the apex. Place a continuous suture from the angle until the hymenal ring. It should include the vaginal mucosa and the rectovaginal fascia. A continuous suture is placed with locking stitches till the hymenal ring. Tie off with knots, to the right, and to the left and finish with another to the right. Cut the thread at 1cm.

* Perineal muscle suture: identify the perineal muscles on both sides of the lesion and approximate them with separate interrupted sutures of “Safil quick” 0/0.

* Skin suture: The end result should have skin approximated but without tension, as a skin suture can increase the incidence of perineal pain in the first postpartum months. Interrupted sutures of “Safil quick” 2/0 are placed, avoiding tension, tying off with three turns as previously described, and cut the thread at 1cm.
